# Revision surgery for symptomatic postoperative pseudocyst following full-endoscopic lumbar discectomy: clinical characteristics and surgical strategies

**DOI:** 10.1186/s12891-022-05791-y

**Published:** 2022-09-03

**Authors:** Bin Zhu, Lanpu Shang, Xiao Han, Xingchen Li, Hongchen Wang, Peiming Sang, Chaoliang Lv, Jian Li, Xiaoguang Liu

**Affiliations:** 1 Department of Orthopaedics, Capital Medical University Affiliated Beijing Friendship Hospital, Beijing, China; 2 Pain Medicine Centre, Peking University Third Hospital, Beijing, China; 3 Department of Spine, Beijing Jishuitan Hospital, Beijing, China; 4 Department of Orthopedics, the First Affiliated Hospital of Zhengzhou University, Zhengzhou, Henan China; 5 Department of Orthopaedics, Beijing Renhe Hospital, Beijing, China; 6 Department of Orthopaedics, Ningbo Medical Center Lihuili Hospital, Ningbo, Zhejiang China; 7 Department of Orthopaedics, Jining NO.1 People’s Hospital, Jining, Shandong China; 8 Department of Orthopaedics, Jinan Central Hospital, Jinan, Shandong China; 9 Department of Orthopaedics, Peking University Third Hospital, 49 Huayuan North Road, Haidian District, 100191 Beijing, People’s Republic of China

**Keywords:** Pseudocyst, Endoscopic discectomy, Postoperative complication, Revision surgery

## Abstract

**Background:**

A symptomatic postoperative pseudocyst (PP) is a cystic lesion that is formed in the operation area of the intervertebral disc, leading to worse symptoms. Some minority patients who developed PP experienced rapidly aggravating symptoms and could not be treated by any kind of conservative treatment. However, no clinical studies have evaluated the clinical characteristics and surgical strategies of symptomatic PP requiring a revision surgery after full-endoscopic lumbar discectomy (FELD). This study aimed to demonstrate the clinical characteristics and surgical strategies of symptomatic PP requiring a revision surgery after FELD.

**Methods:**

We retrospectively analyzed the data of patients who received FELD revision surgeries due to symptomatic PP formation between January 2016 and December 2021. Common characteristics, time intervals of symptom recurrence and revision surgery, strategies for conservative treatment and revision surgery, operative time, imaging characteristics, numeric rating scale (NRS) score, Oswestry disability index (ODI) and overall outcome rating based on modified MacNab criteria were analyzed.

**Results:**

Fourteen patients (males = 10, females = 4), with a mean age of 24.4 years, were enrolled. The mean time intervals of symptom recurrence and revision surgery were 43.5 and 18.9 days respectively. While the patients were conservatively managed with analgesics and physical therapy, pain persisted or progressively worsened. In comparison to the initial herniated disc, the PP was larger in 11 cases, and up- or down-migrated in four cases. The PP location included the lateral recess (*n* = 12), foraminal (n = 1), and centrolateral (n = 1) zones. One of the two cases treated by percutaneous aspiration (PA) was eventually treated by FELD as pain was not relieved. Follow-ups revealed an improved mean NRS score from 7.1 to 1.4, mean ODI from 68.6 to 7.9% and promising overall surgical outcomes.

**Conclusions:**

The progressively severe pain experienced due to PP might be a result of its enlargement or migration to the lateral recess and foraminal zones. As complete removal of capsule is the goal, we recommend FELD instead of PA.

## Introduction

Endoscopic lumbar discectomy has become one of the main surgical methods for lumbar disc herniation (LDH) [1–3]. The formation of postoperative pseudocyst (PP) at the decompression area is a rare postoperative complication after full-endoscopic lumbar discectomy (FELD) surgeries. Most patients who developed PP were asymptomatic or suffered from mild symptoms which could be relieved by conservative treatment [4, 5]. However, a minority experienced rapidly aggravating symptoms and could not be treated by any kind of conservative treatment [4, 6–9]. Herein, we reported a series of revision surgeries for patients with symptomatic PP and unsuccessful conservative treatment in 7 main minimally invasive spine centers of China between 2016 and 2021. The study aimed to investigate the clinical characteristics, mechanism of formation, and surgical strategies for this unintended postoperative complication.

## Materials and methods

This retrospective study was approved by the ethics committee of Peking University Third Hospital, Beijing, People’s Republic of China (S2019312). The study included patients who underwent FELD between January 2016 and December 2021 in 7 main minimally invasive spine centers of China. A transforaminal approach via the Thomas Hoogland endoscopic spine system (THESSYS) technique [10] or an interlaminar approach [11] was employed by 8 surgeons for these surgeries.

The inclusion criteria are: (1) Patients received FELD surgeries due to lumbar disc herniation after unsuccessful conservation treatment for at least 6 weeks; (2) Patients achieved complete remission and returned to normal work; (3) Symptoms reoccurred and magnetic resonance imaging (MRI) revealed typical manifestations: a cystic lesion at the discectomy site, with low intensity on a T1-weighted image (T1WI) and high intensity on a T2-weighted image (T2WI) [7]; (4) All kinds of noninvasive treatment including analgesics, physical therapy, and bed rest were unsuccessful; and (5) Patients eventually received revision surgeries.

The exclusion criteria are: (1) MRI confirmed that the relapse of symptoms was due to disc fragment (recurrence or incomplete decompression); (2) Patients refused the revision surgery.

Common characteristics such as age, gender, level, surgical approach and operative time were recorded. The first surgeries and the endoscopic revision surgeries of the 14 patients performed under local anesthesia (10 mL of 2% lidocaine, 10 mL of 1% ropivacaine, and 20 mL of 0.9% saline solution). Time interval of symptom recurrence, conservative treatment strategy before revision surgery, time interval of revision surgery, surgical strategy of revision surgery, and imaging characteristics were also analyzed. Time interval of symptom recurrence is defined as the duration between the first surgery and recurrence of symptoms. Time interval of revision surgery is defined as the duration between the recurrences of symptoms and the revision surgery. At the last follow-up, the degree of change of the PP was classified as total or near total. Near total regression was defined as a volume decrease of at least 90% on the last MRI. Total regression was defined as completely remove on the last MRI. The clinical outcomes were evaluated using the numeric rating scale (NRS) for pain intensity, Oswestry disability index (ODI) and modified MacNab criteria for the overall outcome, which is classified into excellent, good, fair, or poor.

## Results

More than 4000 FELD surgeries were performed across the seven minimally invasive spine centers of China annually. Fourteen patients were enrolled in the study based on the predetermined inclusion and exclusion criteria, five of whom were treated in our center while the remaining nine were treated in the other six centers.

The demographics, clinical features and surgical outcomes of the patients are shown in Table 1. Of the 14 patients, ten were males and four were females. They have a mean age of 24.4 years (range: 15–59 years). There were three cases of L3–4 level, six cases of L4–5 level, and five cases of L5-S1 level. Thirteen of them were treated by a transforaminal approach and one of them was treated by an interlaminar approach in their previous surgery. The mean operative time of the first surgery was 59.9 min. The time interval of symptom recurrence ranged from 14 to 90 days, with an average of 43.5 days. All the patients were conservatively managed with analgesics, physical therapy, and bed rest. Pain persisted in four cases, and was progressively increased in ten cases during the conservative treatment. The mean NRS before the revision surgery was 7.1. The mean ODI before the revision surgery was 68.6%. The time interval of revision surgery ranged from 5 to 90 days, with an average of 18.9 days. Eleven cases received conservative treatment for less than 1 month, due to rapidly progressive severe radicular pain.

**Table 1:** Demographic features and clinical outcomes of symptomatic postoperative pseudocyst (PP) requiring revision surgery after Full-endoscopic lumbar discectomy (FELD)

No	Age	Gender	Level	Location of herniated lumbar disc	Approach of the first surgery	operative time of the first surgery (min)	Time interval of symptom recurrence	Time interval of revision surgery	Location of PP	Change of PP size	NRS before revision surgery	ODI before revision surgery(%)	Approach of revision surgery	operative time of revision surgery (min)	Follow up (months)	NRS	ODI(%)	PP change at the last follow-up	MacNab criteria
1	20	M	L5-S1	centrolateral	TF	62	90	90	lateral recess and up-migrated	larger	6	60	IL	38	12	1	0	Total	Excellent
2	27	F	L4–5	lateral recess	TF	56	27	5	lateral recess	larger	8	74	TF and TF	31 and 50	2	1	12	Near total	Good
3	18	M	L4–5	lateral recess and down-migrated	TF	49	41	11	lateral recess and down-migrated	larger	7	66	TF	42	2	2	10	Near total	Good
4	17	F	L5-S1	centrolateral	TF	71	14	10	lateral recess	similar	8	70	TF	49	5	0	2	Total	Excellent
5	16	F	L5-S1	centrolateral	TF	60	60	22	lateral recess	larger	6	58	TF	32	5	1	8	Total	Good
6	26	M	L4–5	centrolateral	TF	54	45	5	lateral recess and down-migrated	larger	8	68	TF	40	29	1	6	Total	Good
7	15	M	L3–4	lateral recess and down-migrated	TF	67	30	8	lateral recess and down-migrated	larger	7	72	TF	45	39	0	0	Total	Excellent
8	28	M	L5-S1	lateral recess	IL	44	30	6	lateral recess and down-migrated	larger	8	78	IL	57	31	2	10	Near total	Good
9	31	M	L4–5	centrolateral	TF	53	21	7	lateral recess	similar	6	64	TF	29	30	2	6	Total	Good
10	23	M	L5-S1	centrolateral	TF	77	40	16	lateral recess	larger	8	80	TF	41	2	2	16	Near total	Good
11	29	M	L3–4	centrolateral	TF	46	30	9	foraminal and up-migrated	larger	8	72	TF	33	17	3	10	Total	Good
12	16	M	L4–5	centrolateral	TF	58	60	35	lateral recess	larger	6	58	PA	21	16	2	12	Near total	Good
13	17	M	L3–4	centrolateral	TF	56	66	36	lateral recess	larger	6	64	PA and TF	17 and 43	6	1	4	Total	Excellent
14	59	F	L4–5	centrolateral	TF	85	55	5	centrolateral	similar	8	76	TF	41	11	2	14	Near total	good
Average	24.4					59.9	43.5	18.9			7.1	68.6		40.8^a^	14.8	1.4	7.9		
Median (Q1 - Q3)	21.5(17, 28)					57(52,68)	40.5(30, 60)	9.5(6, 22)			7.5(6, 8)	69(63,74.5)		41(32.75,36.75)^a^	11.5(5, 29)	1.5(1,2)	9(3.5,12)		

*PA* percutaneous aspiration, *TF* transforaminal, *IL* interlaminar, *ODI* Oswestry Disability Index; ^a^Average and Median (Q1 - Q3) of operative time of revision surgery were calculated with endoscopic surgery except PA

**Fig. 1:**
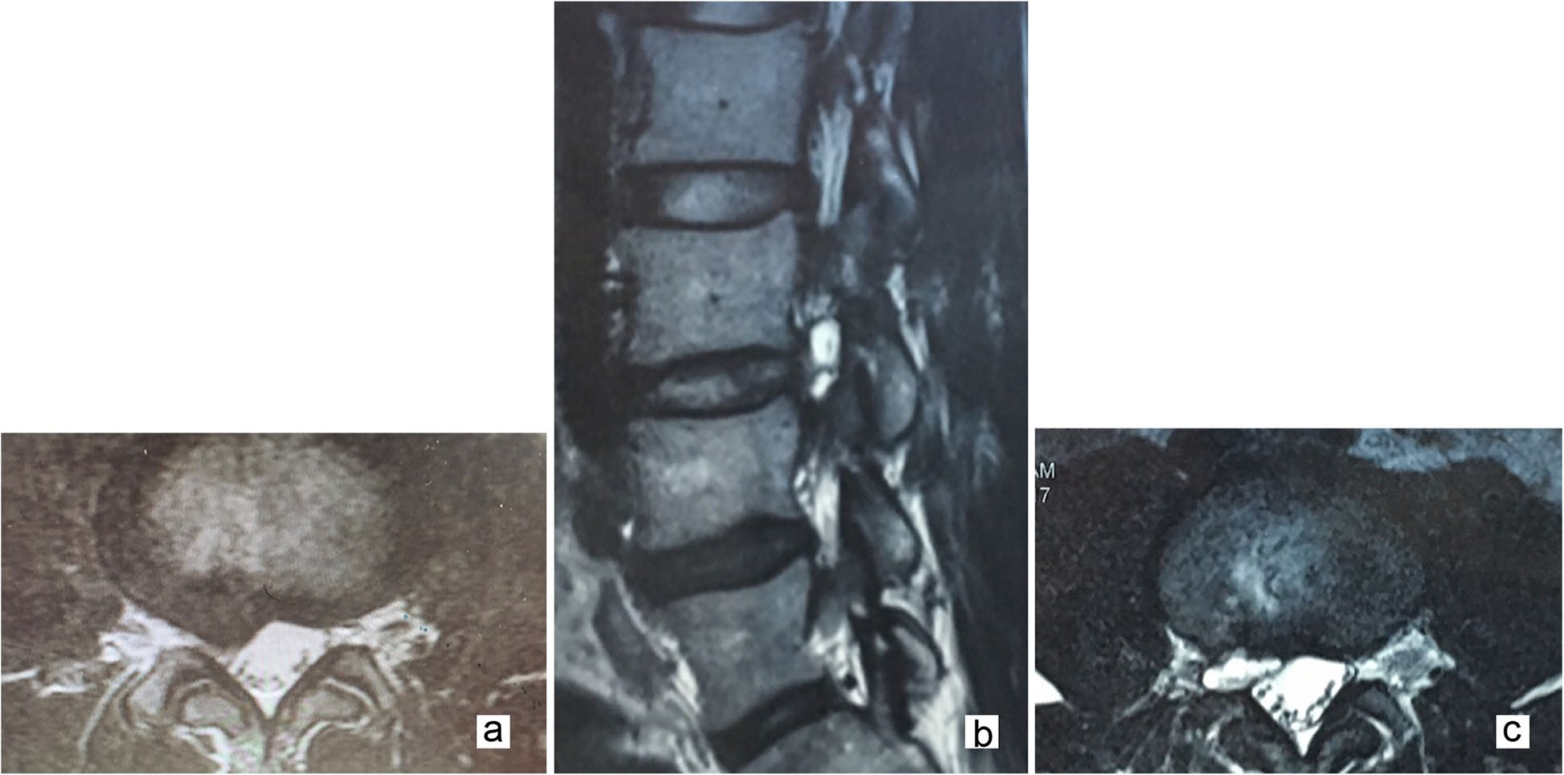
Case illustration of a symptomatic postoperative pseudocyst requiring full-endoscopic lumbar discectomy (Case 11). Axial MRI examination of a 29-year-old male patient on a T2-weighted image (T2WI) showing a right-sided disc herniation at L3–4 level (**a**). MRI revealing a cystic lesion at the discectomy site (**b**, **c**)

From their MRI images, 11 patients had PP that was larger than their initial herniated lumbar disc. Four cases had up- or down-migration of the PP in relation to the initial herniated lumbar disc. Eight cases had their PP migrated from the centrolateral zone of their herniated lumbar disc to the lateral recess zone. The PP of case 11 migrated to the foraminal zone of the spinal canal (Fig. 1).

Thirteen patients received an endoscopic revision surgery, 12 of whom were treated using a transforaminal approach and one of whom was treated using an interlaminar approach. The mean operative time of the endoscopic revision surgery was 40.8 min. Endoscopic revision surgery was initially planned for case 12 as well, but was halted after 2 ml of yellow fluid was drained and the symptoms of the lower limbs were relieved following the puncture of the intervertebral foramen during the surgical process. On the contrary, the symptoms of case 13 were not relieved after percutaneous C arm-guided aspiration and hence, endoscopic revision surgery was finally performed. The cases were followed up for an average of 14.8 months. The NRS score improved from 7.5 (Q1: 6, Q3: 8) to 1.5 (Q1: 1, Q3: 2) after the revision surgery. The ODI improved from 69%(63,74.5%) to 9%(3.5,12%). At the last follow-up, 8 cases showed total regression and 6 cases showed near total regression. There were no nerve injury, dural tear and other complications after surgical treatment. The final outcome, based on the modified MacNab criteria, was found to be excellent in four patients and good in ten patients post revision surgery.

**Fig. 2:**
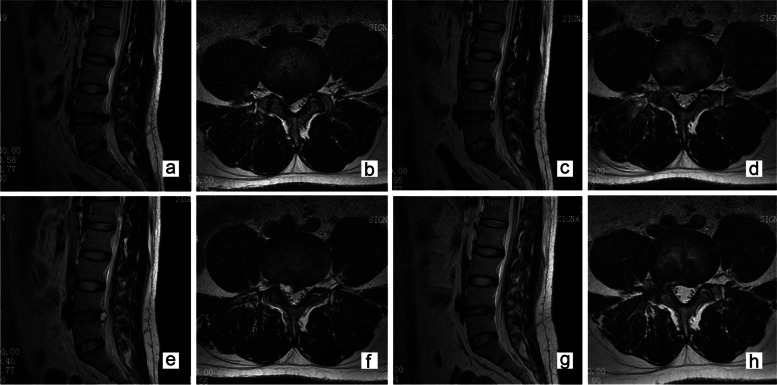
Case illustration of a symptomatic postoperative pseudocyst requiring full-endoscopic lumbar discectomy (Case 9). Sagittal and axial MRI of a 31-year-old male patient on a T2-weighted image (T2WI) showing a right-sided disc herniation at L4–5 level (**a**, **b**). MRI revealing a complete decompression 3 days after FELD (**c**, **d**). MRI demonstrating a cystic lesion at the discectomy site (**e**, **f**). MRI confirming that the cyst was removed during a follow-up 2 months after the revision surgery (**g**, **h**)

Typical case scenario 1 (Case 9): A 31-year-old male patient was presented with a right-sided disc herniation at L4–5 level. MRI was performed to confirm complete decompression 3 days after FELD. Radicular pain recurred (NRS score = 6) 21 days post FELD, and the conservative treatment that was given was ineffective for a week. MRI revealed a cystic lesion at the discectomy site. Revision surgery was eventually performed (Fig. 2).

**Fig. 3:**
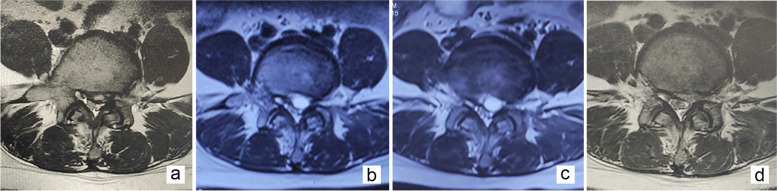
Case illustration of a symptomatic postoperative pseudocyst (PP) requiring full-endoscopic lumbar discectomy (Case 2). Axial MRI of a 27-year-old female patient on a T2-weighted image (T2WI) showing a left-sided disc herniation at L4–5 level (**a**). Radicular pain recurred (NRS score = 8) 27 days post FELD, and the conservative treatment that was given was ineffective for 5 days. MRI revealing a cystic lesion at the discectomy site (**b**). MRI demonstrating PP re-formation at the same site 10 days after the first revision surgery (**c**). MRI confirming that the PP was removed during a follow-up 1 day after the second revision surgery (**d**)

Typical case scenario 2 (Case 2): A 27-year-old female patient was presented with a left-sided disc herniation at L4–5 level. We performed two revision surgeries, 14 days apart. In the first revision surgery, we found severe adhesion and only used radio-frequency electrocautery to puncture and take out part of the capsule. As the patient felt a complete relief of pain following the removal, the surgery was concluded. However, the patient suffered from a similar pain again after 10 days. MRI demonstrated a PP formation at the same site. As such, the second revision surgery was done with a fully exposed operative field and the capsule was completely removed. A follow-up 2 months later showed that the pain that was experienced had improved (Fig. 3).

## Discussion

Very few studies report on the formation of symptomatic PP at the same surgical level. Hence, we did a PubMed search for “lumbar cystic lesion”, “lumbar discal cyst”, “postdiscectomy pseudocyst”, “postoperative annular pseudocyst” or “postoperative pseudocyst” to review the different studies on symptomatic PP and their surgical outcomes on March 2022 (Table 2). From the search results, it was revealed that pseudocyst formation has been reported after open discectomy, microdiscectomy, and microendoscopic discectomy [6, 9, 14–16]. Kang and Park reported an approximately 1% prevalence of PP formation after endoscopic lumbar surgery [4]. However, a reporting bias may be present as all the patients in their study were young, male soldiers. In China, postoperative MRI was not performed for every patient. As such, the patients with mildly symptomatic or asymptomatic PP may not be documented. Therefore, the prevalence of PP is suspected to be higher than what we have observed in our study.

**Table 2:** Studies of symptomatic postoperative pseudocyst (PP) with their respective outcomes

Study	No. of pts	Age	Gender	Level	Primary procedure	Time of symptom recurrence	Management	Follow-up duration	Clinical Outcomes
Li J et al.(2021) [12]	1	30	M	L4–5	PELD	37 days	ozone ablation	1 year	Pain improved
Xu W et al.(2021) [13]	1	27	M	L5-S1	PEID	40 days	open cyst resection	6 months	Symptoms were significantly relieved
Manabe et al. (2019) [8]	1	21	M	L4–5	PELD	6 weeks	PELD after failure of injection	5 days	Pain improved
Shiboi et al. (2017) [7]	2	27 14	1 M 1 F	2 L4–5	2 PELD	20 days 30 days	1MED 1PELD	29 months 2 months	Pain improved
Prasad and Menon (2017) [9]	1	30	M	L4–5	MD	25 days	Surgery (L5 laminectomy and right-sided medial facetectomy)	17 months	Excellent
Jha et al. (2016) [14]	2	16 18	1 M 1 F	L4–5 L5-S1	2 MED	1 week for both	2 Conservative	6 months	No residual symptoms
Yu et al. (2016) [15]	1	27	M	L4–5	Open discectomy	About 2 weeks	C-arm guided aspiration/injection	3 months	Pain improved
Chung et al. (2012) [6]	12	29.3 ± 11.9 (20–57)	1 F 11 M	3 L3–4 7 L4–5 2 L5-S1	9 MD 3 PELD	Average 23.3 days (9–38 days)	5 MD 1 Aspiration 6 Conservative	17–300 days	10 Excellent 2 Good
Kang and Park (2011) [4]	15	22.6 ± 5.8 (18–55)	15 M	6 L4–5 9 L5-S1	15 PELD	Average 53.7 days (11–118 days)	1 PHL 4 PELD 10 Conservative	24.8 ± 16.5 months	The results between conservative treatment and surgical treatment were of no significant differences.
Young et al. (2009) [16]	2	60 30	2 M	1 L4–5 1 L5-S1	2 MD	1 month 8 months	1 Conservative 1 CT-guided aspiration/injection	4 years 17 months	Able to participate in occupation or daily activities

*CT* computed tomography, *MD* microdiscectomy, *MED* microendoscopic discectomy, *PELD* percutaneous endoscopic lumbar discectomy, *PEID* percutaneous endoscopic interlaminar discectomy, *PHL* partial hemilaminectomy and discectomy, *pts* patients

### Clinical characteristics

The characteristics of PP seemed to be similar to those of discal cysts as reported [17]. In all the reviewed studies, PP was found to be more common in young male patients at L4–5 level. The same could be observed for this study in general. The highest prevalence of herniated lumbar disc was among people aged 30 to 50 years, with a male to female ratio of 2:1 [18]. In the people younger than 35 years, the male to female ratio was significantly higher than that of other age groups, and men were more likely to suffer from lumbar disc herniation. In this study, we found that the male to female ratio of PP is 2.5:1, which was similar to the prevalence rate of lumbar disc herniation. More male patients may have more PP cases. The diagnosis and differential diagnosis of symptomatic PP mainly depend on clinical manifestations and imaging scans. The 14 cases that ended up with revision surgeries had similar disease progression: (1) Most of them were young male patients with a definite diagnosis of lumbar disc herniation, and the clinical manifestations were related to compression of nerve root(s) by the herniated disc. All patients received FELD surgeries; (2) The symptoms that the patients experienced were alleviated after the first surgery; (3) Radicular pain of lower extremity, similar to that experienced before surgery, reoccurred after 40.5 (Q1: 30, Q3: 60) days without obvious inducement; (4) Severe pain median NRS score = 7.5 (Q1: 6, Q3: 8) persisted, or the pain was progressively affecting normal life. The degree of pain that the some of the patients felt during the recurrence was higher than that before surgery, and the conservative treatment that they received was ineffective; (5) MRI imaging revealed the formation of a cystic lesion at the discectomy site, with low intensity on a T1WI and high intensity on a T2WI, that was compressing the dural sac and nerve root. PP exhibited the following characteristics, in relation to that of the initial herniated lumbar disc: larger, up- or down-migrated, located in lateral recess or foraminal zone; (6) The patients received revision surgeries within a very short period of time median = 9.5 (Q1: 6, Q3: 22) days; (7) Most of the surgical outcomes were satisfactory. In contrast to lumbar discal cyst with spontaneous regression [5, 19], 13 out of 14 of the PP cases in our study exhibited at least one of the following three characteristics: larger, up- or down-migrated, located in lateral recess or foraminal zone; which might lead to severe compression of the dural sac and nerve root. As the pain progressed rapidly due to the severe compression and the patients did not respond to conservative treatment, revision surgery was eventually performed.

The common clinical manifestation of PP is postoperative recurrent radicular pain, which is similar to that of the recurrence of disc herniation [20]. As such, MRI is required for a differential diagnosis. While a disc herniation is presented with a low intensity on both T1WI and T2WI, a PP is typically presented with a high signal, clear boundary and cystic change on a T2WI. However, it may not always hold true [21]. Hence, intraoperative identification is also important. Generally, PP has a dark-red capsule without disc fragments or hematoma. While the MRI scan of case 14 revealed a well-defined cystic lesion at L4–5 level, the cystic lesion was presented with a medium signal on a T2WI. This is different from those of the other patients. In the endoscopic view of case 14, the surgeon found the new prolapsed disc tissue inside the cyst wall, thereby suggesting that the PP might consist of not only cystic tissue but also disc components. Shiboi et al. [7] reported a case of recurrent LDH that mimicked a symptomatic PP. Hence, such cases should be considered carefully to rule out LDH recurrence. This might also be the reason why this case was treated with a revision surgery despite the absence of the three aforementioned characteristics of PP.

### Pathology and pathogenesis

**Fig. 4:**
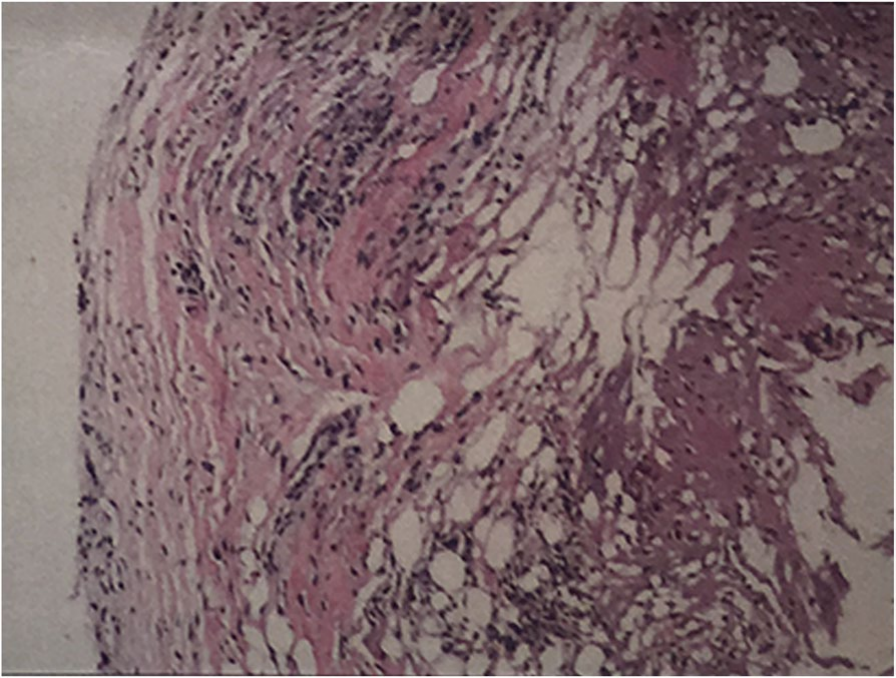
The pathological results of the cystic wall (Case 8): degenerative fibrocartilage and loose fibroadipose tissue accompanied by inflammatory cell infiltration, focal hemorrhage, mucus degeneration and cystic change

In all the reviewed studies, the pathology and pathogenesis of PP were still unclear. Young et al. hypothesized that granulation tissue may form a pseudocapsule around the herniated disc fragment [16]. If the disc fragment is removed without disrupting the pseudocapsule, fluid may accumulate and PP may be formed. The pathological results of the cystic wall indicated that its main component is fibrous connective tissue (Fig. 4), which might be the result of an inflammatory response of the connective tissue due to postoperative annular defects. As most of the patients in our study are young, the strong ability of granulation or fibrous tissue formation as well as self-repair of annular fibrosus might be the cause of symptomatic PP formation. Male patients may return to work and resume daily activities earlier than female patients, and in the early postoperative period, the increased pressure on the intervertebral disc may promote the formation and enlargement of PP. However, a large cohort is needed to prove this hypothesis. Additionally, the inadequate treatment and resection of the annular fibrosus might have led to the formation of the pseudocyst. Together with that, an insufficient resection of the facet joint might have limited the opening of the spinal canal thereby leading to symptomatic PP. It has been reported that the interlaminar approach, in comparison to the transforaminal approach, was correlated with PP formation [4]. However, the transforaminal approach was performed in 13 out of 14 cases in this study. This implies that the surgical approach may not be significantly related to the formation of PP.

### Management strategies

In all the reviewed studies, conservative treatment, C arm/computed tomography (CT)-guided aspiration and/or injection, microdiscectomy, microendoscopic discectomy, and PELD had been used to treat the patients for PP. Clinical and radiological recovery of the symptomatic lumbar discal cyst could only be obtained through conservative therapy [5, 19, 22]. In our cases with severe pain caused by the compression of dural sac or nerve root by cystic lesions as confirmed by MRI, however, conservative treatment was ineffective, and revision surgery was required. It should be noted that, the patient in Case 12 felt a relief of pain after the puncture of the intervertebral foramen and did not undergo endoscopic revision surgery, thereby suggesting that percutaneous aspiration (PA) could be an alternative treatment with less trauma [6, 15, 16]. However, C arm/CT-guided aspiration and/or injection cannot remove the capsule wall, thereby risking the recurrence of PP. [8] Furthermore, it will also be difficult to aspirate completely if the PP is a multicystic cavity. This was evident in Case 13, where the pain was not improved after PA and the patient eventually underwent an endoscopic revision surgery.

**Fig. 5:**
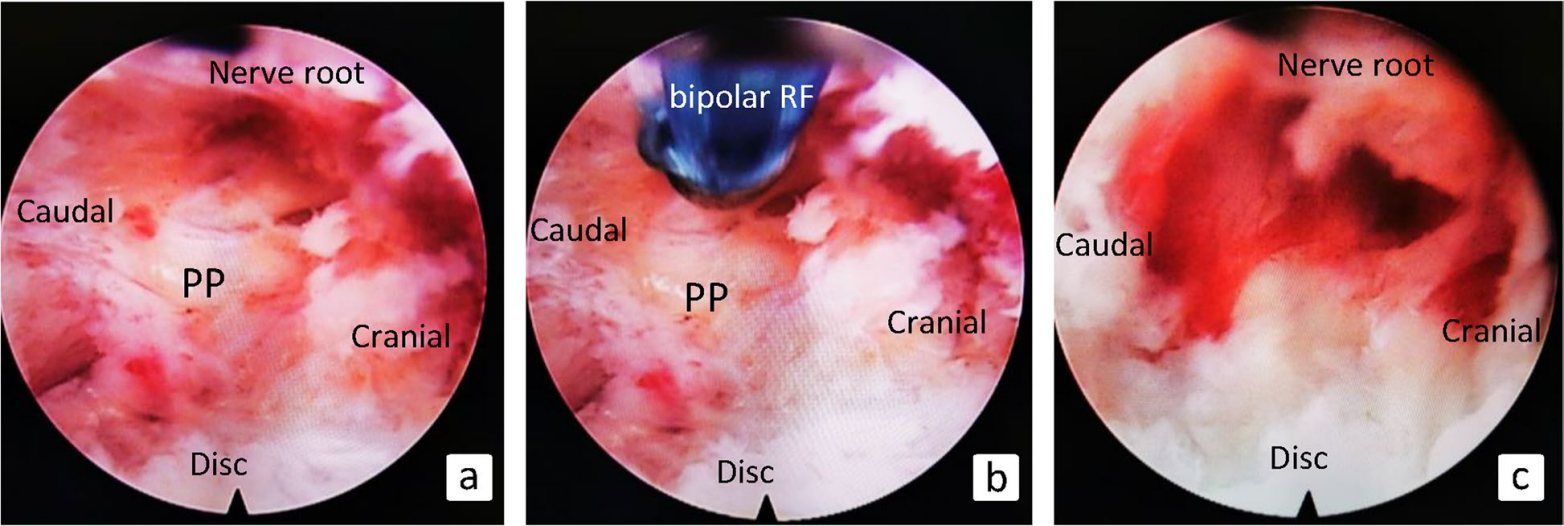
The endoscopic view (Case 3): Show severe adhesion surrounding the PP (**a**). Use bipolar radiofrequency (RF) to separate the PP from the nerve root carefully (**b**). PP was removed and annuloplasty was done using radiofrequency coagulation (**c**)

Endoscopic lumbar discectomy has been reported for the treatment of discal cysts [23–25]. In this study, FELD was performed for the revision surgeries of 13 cases and the postoperative recoveries were satisfactory. The mean operative time of revision surgery was less than that of the first surgery. Because we operated through the original surgical path in the revision surgery, there was no need for facetectomy and discectomy again. In the endoscopic view, we generally found severe adhesion surrounding the PP (Fig. 5), dural sac, ligamentum flavum, and disc material, as well as well-developed vasculatures which causes bleeding when removing PP. Hence, we should separate the adherent tissues carefully and remove as much of the capsule of PP as possible during the revision surgery to minimize having to perform another revision surgery. This is evident in Case 2, where we performed two revision surgeries with a 14 days interval. If it is difficult to completely resect the cyst, the potential for recurrence may be reduced by annuloplasty using radiofrequency coagulation [8].

### Limitation of the study

This study had some limitations. The exact total number of patients treated in 7 minimally invasive spine centers is not available. Therefore, the prevalence of PP requiring revision surgery is not measured correctly. In addition, the lack of surgeon standardization is another limitation. The data is collected from 8 surgeons. A further clinical study will be needed to figure out the definite pathology and pathogenesis of PP.

## Conclusion

Symptomatic PP was mostly found in young male patients. Surgeons should distinguish it from a recurrent disc herniation. The implicated patients who require a revision surgery often experience a severe pain that is rapidly progressive. In addition, the PP in these patients possesses at least one of the following three MRI characteristics: larger, up- or down-migrated, located in lateral recess or foraminal zone. It is important for surgeons to remove as much of the capsule as possible during the revision surgery to prevent the recurrence of PP. As such, it is recommended to perform revision surgery such as FELD, instead of PA. The pathology and pathogenesis of PP remain unclear, but may be related to factors such as insufficient resection of the annular fibrosus and facet joint during the first surgery.

## Data Availability

The datasets used and/or analyzed during the current study are available from the corresponding author upon reasonable request.

## Abbreviations

PP: Postoperative pseudocyst
FELD: Full-endoscopic lumbar discectomy
NRS: Numeric rating scale
PA: Percutaneous aspiration
LDH: Lumbar disc herniation
THESSYS: Thomas Hoogland endoscopic spine system
MRI: Magnetic resonance imaging
TF: Transforaminal
IL: Interlaminar
CT: Computed tomography
MD: Microdiscectomy
MED: Microendoscopic discectomy
PELD: Percutaneous endoscopic lumbar discectomy
PEID: Percutaneous endoscopic interlaminar discectomy
PHL: Partial hemilaminectomy and discectomy
Pts: Patients
